# Structure of preantral follicles, oxidative status and developmental competence of in vitro matured oocytes after ovary storage at 4 °C in the domestic cat model

**DOI:** 10.1186/s12958-018-0395-1

**Published:** 2018-08-10

**Authors:** Anna Rita Piras, Giovanni Pietro Burrai, Federica Ariu, Laura Falchi, Maria Teresa Zedda, Salvatore Pau, Sergio Domenico Gadau, Elisabetta Antuofermo, Daniela Bebbere, Sergio Ledda, Luisa Bogliolo

**Affiliations:** Department of Veterinary Medicine, University of Sassari, Via Vienna 2, 07100 Sassari, Italy

## Abstract

**Background:**

Storage conditions during transportation of explanted ovaries are a critical step in setting up fertility preservation protocols in both animal and human fields. Here, we evaluated the effects of ovary storage at 4 °C on the preservation of preantral follicles and oocytes retrieved from antral follicles using the domestic cat as model.

**Methods:**

Ovaries were harvested from fifty-five healthy domestic queens during ovariectomy and stored at 4 °C for 0 (control), 24, 48, 72 and 96 h. In Experiment 1, the effects of the storage period at 4 °C on the morphology, cytoskeleton (α/β tubulin) and DNA integrity (phosphorylation of histone H2AX) of preantral follicles were investigated. In Experiment 2, oocytes recovered from antral follicles were matured and fertilized in vitro to evaluate their meiotic and developmental competence*.* Reactive oxygen species (ROS), glutathione (GSH) and lipid peroxidation were measured in matured oocytes.

**Results:**

The results showed that: a) storage up to 24 h did not affect the morphology and the DNA integrity of preantral follicles; b) extended storage times caused progressive morphological abnormalities, disassembling of microtubules and DNA damage; c) storage up to 48 h did not influence in vitro meiotic maturation of oocytes nor cleavage after in vitro fertilization. However, only oocytes stored within the ovary for 24 h produced blastocysts in a percentage similar to control oocytes; d) GSH levels of in vitro matured oocytes did not change at any time during ovary storage; a progressive increase in ROS levels was detected from 48 h associated with elevated lipid peroxidation at 72 and 96 h of storage.

**Conclusions:**

Storage of cat ovaries for up to 24 h caused minimal alteration of preantral follicles and oocytes. The extension of the storage period beyond 24 h progressively impaired the structure of follicles, and modified the oxidative status of in vitro matured oocytes and their developmental competence after in vitro fertilization. This information may help when setting up programs for fertility conservation, especially for wild feline species which die in geographic areas located far away from ARTs centers.

## Background

Most of the 36 living species of wild cats are formally listed as threatened by extinction mainly due to poaching, habitat destruction and infectious diseases [1]. Assisted Reproductive Technologies (ARTs) contribute to the preservation of endangered feline species. In domestic felids considerable progress has been made in the development of modern methodologies of ARTs including in vitro embryo production and banking of genetic resources. Some of these techniques have been included in programs for the conservation of feline wildlife species [2, 3].

The possibility of rescuing germplasm from feline females after sudden death in their natural habitat or zoos or after medical ovariohysterectomy may at times require the shipment of ovaries for long distances, from the retrieval site to the ART centres. Therefore, storage conditions (temperature, duration, medium) during transportation of explanted ovaries are a critical step in setting up of fertility preservation protocols.

In this context, studies conducted to establish the appropriate transportation temperature for ovaries provided evidence that feline oocytes, in contrast to other species, have a unique tolerance to cold storage at 4 °C [4–6]. A pivotal study by Johnston et al. [7] found that oocytes from rare wild species could be matured and fertilized in vitro following ovary storage for as long as 36 h in cold (4 °C) phosphate buffer saline (PBS). Histological evaluation of cold stored ovaries of domestic cats further supported the concept that immature oocytes have some degree of cold tolerance [8]. Other studies reported unaffected in vitro maturation (IVM) rates when domestic cat ovaries were stored at 4 °C up to 72 h. However, only oocytes stored within the ovary for 24 h could produce blastocysts after in vitro fertilization [9] and retained competence to develop into live offspring after transfer into recipients [10]. More recently, findings by Luu et al. [11] indicated that the addition of relaxin to the IVM medium improved the rate of blastocyst development of cat oocytes from ovaries stored for 24 h at 4 °C. Furthermore, superoxide dismutase (SOD) supplementation to ovary transportation medium enhanced in vitro embryo production from oocytes of domestic cat ovaries preserved at 4 °C for up to 72 h [12].

Most studies on the cold storage of feline ovaries have focused on the preservation of oocytes from antral follicles. This is a limitation as an ovary contains a large amount of preantral follicles, which represents the female’s ovarian reserve and includes more than 90% of the follicular population [13]. Therefore, the pool of preantral follicles constitutes a potentially rich source of genetic material which could be used in fertility preservation programs for genetically valuable animals, including females of endangered species that die before reaching sexual maturity [14]. In fact, the ovarian cortex can be cryopreserved and utilized for xenotrasplantation or, alternatively, preantral follicles can be isolated and cultured in vitro until maturity. Slow freezing of cat ovarian cortex in conjunction with xenotransplantation was first reported by Bosch et al. [15]. Moreover, mature oocytes have been obtained after long-term transplantation of ovarian cortex from female lions into immunodeficient mice [16]. In several animal species, including cats, attempts have been made to develop in vitro culture systems for the growth of preantral follicles with variable results [17–20]. Full term culture of primordial follicles to a mature stage and birth of viable offspring after in vitro fertilization and embryo transfer have only been achieved in mice and were first described by Eppig and O’Brien [21].

Nowadays, to the best of our knowledge, there is little information about the impact of cold storage on the health status of preantral follicles of feline ovaries and on the maximal tolerable storage duration [22]. Evidence from other animal models indicates that cold temperature favors the preservation of preantral follicles [23–27].

The storage of ovarian tissue during transportation has great importance not only for rare and endangered species, but also for humans. Indeed, transportation of human ovarian tissue from cancer patients to a single central national center is conducted in several countries [28]. In Denmark [29, 30] Germany, Switzerland, Austria [31, 32] and in the United States [33–36] ovarian tissue is cryostored only in specific centers and finding the optimal transportation conditions will probably also be a benefit to such centers.

In the present study, the domestic cat was used as an experimental model to evaluate the preservation of preantral follicles and of oocytes retrieved from antral follicles in ovaries stored at 4 °C for different time periods. Therefore, we analyzed: a) morphology, microtubular cytoskeleton and DNA damage in preantral follicles and, b) developmental competence of oocytes after in vitro maturation and fertilization as well as oxidative status in matured oocytes.

## Methods

### Chemicals

All chemicals, in this study, were purchased from Sigma Chemical CO. (St. Louis, MO, USA) unless stated otherwise.

### Ovary collection and storage

Ovaries were harvested from fifty five healthy, domestic queens (*Felis catus*, 8 months-2 years of age) at random stages of their oestrus cycles during routine ovariectomy at the Veterinary Teaching Hospital of the University of Sassari (Italy). The ovaries were placed in sterile 15-ml tubes containing Phosphate Buffered Saline (PBS, cat n°18,912–014, Gibco, Life Technologies, Carlsbad, California, USA) with penicillin (100 mg/L), streptomycin (100 mg/L) [8, 12]., Calcium Dichloride Hydrate (CaCl_2_H_2_O, 133 mg/L) and Magnesium Chloride Hexahydrate (MgCl_2_•6H_2_O, 100 mg/L). The divalent cations, calcium and magnesium, were added to the PBS solution in order to preserve cellular adhesion and tissue integrity in the ovary during storage at 4 °C [37, 38]. The ovaries were immediately transported to the laboratory at room temperature (22–25 °C) and randomly divided in two groups: fresh ovaries (0 h, control group) and ovaries to be stored at 4 °C (stored group). The ovaries from each storage group were transferred under sterile conditions to a 15-ml tube containing 4 ml of fresh pre-cooled PBS and held at 4 °C in a refrigerator for different lengths of times (24, 48, 72, 96 h). Control and stored ovaries were processed according to the following experimental design.

### Experimental design

The experimental design includes two experiments. Experiment 1 was performed to evaluate the effect of different storage times (0, 24, 48, 72 and 96 h) at 4 °C on the morphology, organization of microtubular cytoskeleton and DNA integrity of preantral follicles.

In Experiment 2, the in vitro meiotic and developmental competence of oocytes collected from antral follicles of control and stored ovaries were evaluated. Moreover, the oxidative status of in vitro matured oocytes from each experimental group was analyzed by quantifying the intracellular levels of Reactive Oxygen Species (ROS), Glutathione (GSH) and lipid peroxidation.

### Experiment 1

#### Morphological evaluation of preantral follicles

Both control and stored ovaries were fixed in Bouin’s solution for 12 h, embedded in paraffin, serially sectioned (5 μm), and mounted onto microscope slides. The sections were either stained with Hematoxylin and Eosin (HE) or used for immunohistochemistry, as described below. In order to evaluate follicular morphology, three sections from each sample were examined. Preantral follicles were classified according to their developmental status as: i) primordial with one layer of flattened cells; ii) primary with one layer of cuboidal cells; iii) secondary with two or more layers of cuboidal cells and a theca cell layer [39]. Moreover, they were graded according to their morphology: structurally normal follicles presented a spherical oocyte with a non-pyknotic nucleus surrounded by well-arranged granulosa cells, while abnormal follicles were characterized by contraction and clumping of oocyte chromatin surrounded by disorganized granulosa cells. To avoid double counting in adjacent sections, the follicles were only analyzed when an oocyte nucleus was present.

#### Fluorescent immunoistochemistry

The histological sections were immersed for 20 min in a 98 °C preheated solution (WCAP, citrate pH 6, BiOptica, Milan, Italy) which simultaneously allows dewaxing, rehydration and antigen unmasking. Slides were mounted in a sequenza chamber (Shandon, Runcorn, UK) before performing immunostaining.

#### Assessment of microtubular cytoskeleton organization

Microtubules are ubiquitous cytoskeletal structures that are formed by the polymerization of α and β tubulin heterodimers [40]. Accordingly, we used monoclonal anti α and β tubulin antibodies to detect the effect of cold storage on the microtubular cytoskeleton organization of ovarian tissue.

Ovarian sections were incubated overnight at 4 °C with a mixture of mouse monoclonal anti α-tubulin (1:1000, Sigma Chemical CO, St. Louis, MO, USA; Cat n° T5168) and mouse monoclonal anti β-tubulin (1:100; Sigma Chemical CO, Cat n° T-7941) antibodies then labeled with donkey anti-mouse Alexa Fluor 488 (1:100, Cat n° A21202, Life Technologies, Invitrogen, Carlsbad, California, USA) for 1 h at room temperature. These specific primary antibodies were chosen for immunofluorescence due to their high affinity to α and β tubulin as indicated by the manufacturer and other Authors [41–44].

The nuclei were counter-stained with Hoechst 33,342 (10 μg/ml; Cat n° B2261) in 1:1 (*v*/v) glycerol/PBS solution. The sections were covered with coverslips, sealed with nail polish, and kept at 4 °C in the dark until observation. Images were acquired by a laser-scanning confocal fluorescence microscope (Leica TCS SP5), equipped with 543 nm HeNe, 488 nm Argon and 405 nm 405-diode laser using an oil immersion 40× objective (NA = 1,25). The parameters related to fluorescence intensity (laser energy, gain, offset and pinhole size) were maintained at constant values during all image acquisitions.

#### Detection of histone H_2_AX phosphorylation

We investigated whether cold storage induced DNA damage in preantral follicles, examining histone H_2_AX phosphorylation (γH_2_AX), a marker of DNA double-strand breaks (DSBs) [45]. The ovary sections were incubated overnight at 4 °C with mouse monoclonal anti-γH_2_AX (phospho S139) antibody (Cat n° ab26350, Abcam, Cambridge; UK) and labeled with donkey anti-mouse Alexa Fluor 488 (1:100) for 1 h at room temperature. The nuclei were counter-stained with propidium iodide (1 μg/ml, Cat n°4170) in 1:1 (*v*/v) glycerol/PBS solution. Images were acquired as described above.

### Experiment 2

#### Oocyte recovery and in vitro maturation

Ovaries from control and stored groups were sliced with a scalpel blade to release the cumulus-oocyte complexes (COCs). The COCs were collected in sterile Petri dishes in dissection medium (DM; 25 mM Hepes-buffered TCM 199) supplemented with 0.1% (*w*/*v*) polyvinyl alcohol (PVA) and antibiotics (100 μg/ml penicillin and streptomycin). Only the oocytes with darkly pigmented ooplasm and completely surrounded by at least one layer of cumulus cells were selected for in vitro maturation (IVM). The COCs were matured in groups of 25–35 in 650 μl of TCM 199 supplemented 0.36 mM pyruvate, 2 mM glutamine, 2.2 mM calcium lactate, 1.2 mM cysteine, 4 mg/ml BSA fatty acid free and FSH 1 IU / ml and LH 1 IU / ml (Pluset; Bio98, Milan, Italy) under mineral oil, in 4-well dishes (Nunc Cell Culture, Thermo Fisher Scientific, Waltham, Massachusetts, USA) in a humidified atmosphere of 5% CO_2_, at 38.5 °C for 24 h [46].

#### Assessment of oocyte nuclear maturation

After IVM, groups of oocytes were completely denuded of granulosa cells via gentle pipetting with a fine bore glass pipette in DM, stained with Hoechst 33,342 (10 μg/ml) in 1:1 (*v*/v) glycerol/PBS solution, placed on a slide and overlaid with a coverslip supported by four droplets of vaseline. The nuclear configuration was classified under an epifluorescent microscope (Olympus IX 70, Italy) as germinal vesicle (GV), germinal vesicle breakdown (GVBD), metaphase I (MI), or metaphase II (MII). The oocytes with the diffusely stained cytoplasm typical of nonviable cells and those with unidentifiable or invisible chromatin were classified as degenerated (Deg).

#### Measurement of intracellular ROS and GSH levels

Groups of matured oocytes (MII) from all experimental groups were selected on the basis of the presence of the first polar body and sampled for intracellular Reactive Oxygen Species (ROS) and Glutathione (GSH) level measurement [47].

Briefly, 2′7′-dichlorodihydrofluorescein diacetate (H2DCFDA, Cat n° D 6883) and 4-chloromethyl-6,8-difluoro-7-hydroxycoumarin (CMF2HC, Cell Tracker Blue*,* Cat n° C12881, Molecular Probes, Oregon, USA) were used to detect intracellular ROS as green fluorescence and GSH level as blue fluorescence. A total of 30–35 oocytes from each treatment group were incubated in the dark for 30 min at 38.5 °C in 5% CO_2_ in air in PBS-PVA containing 10 μM H_2_DCFDA or 10 μM CellTracker Blue. After incubation, the oocytes were washed with PBS-PVA, placed in 50 μL droplets, and observed using an epifluorescence microscope (Olympus IX 70, Italy) with UV filters (460 nm for ROS and 370 nm for GSH). The oocytes were positioned in the plane of focus, and the area of measurement was adapted to the size of the oocyte. Microscope adjustments and photomultiplier settings were kept constant for all experiments. The data of emission intensity/oocyte were reduced by compensation for the background fluorescence. The fluorescent images were saved as graphic files in. TIFF format. Fluorescence intensitiy was analyzed with Image J software (version 1.40; National Institute of health, Bethesda, MD). Results were normalized to control values for each experiment. The experiments were performed in triplicate.

#### Lipid peroxidation assay

Lipid peroxidation assay was performed with 4, 4-difluoro-5- (4- phenyl-1, 3-buttadienyl)-4-bora 3a, 4a-diaza-s-indacene-3-undecanoic acid 581/591C11 (BODIPY D3861, Life Technologies, Invitrogen, Carlsbad, California, USA) [48]. Briefly, a total of 30–35 MII oocytes recovered from the different groups were incubated with 10 mM BODIPY stain for 30 min at 38.5 °C in 5% CO_2_ in air. The dye loaded oocytes were then washed twice in DM and mounted on slides. Green and red fluorescence of BODIPY were determined using a confocal laser scanning microscope equipped with Argon and Helium/Neon lasers at excitation wavelengths of 488 and 543 nm and emission spectra of 500–530 nm (green) and greater than 560 nm (red). Levels of lipid peroxidation were measured by the ratio of green to red fluorescence. Results were normalized to control values for each experiment. The experiments were performed in triplicate.

#### In vitro fertilization and embryo culture

Spermatozoa were collected from epididymides of cats following routine orchiectomy [49, 50] and frozen according to the procedure described by Tsutsui et al. [51]. Before IVF, straws of semen were thawed by immersion in a 37 °C water bath for 30s and centrifuged at 620 x g for 5 min. The supernatant was discarded and the pellet was re-suspended in synthetic oviductal fluid (SOF) containing 6 mg/ml BSA, 50 μg/ml gentamicin (IVF medium). After maturation, the COCs were co-incubated with 1 × 10^6^ motile spermatozoa/mL in IVF medium at 38.5 C in 5% CO_2_ in air with maximum humidity [52].

After 22 h, the presumptive zygotes were washed and cultured in SOF containing 4 mg/ml BSA, 100 IU/ml penicillin and 1% MEM non-Essential amino acids. On day 3 after IVF (day = 0) the embryos were transferred to SOF supplemented with 10% fetal calf serum and 2% MEM essential amino acids [52]. On day 2 and day 7 of IVC, respectively, the number of embryos cleaved and developing to blastocyst stage was determined. Blastocysts were stained with Hoechst 33,342 (10 μg/mL), mounted on a glass slide and examined under epifluorescent microscope (Olympus IX70, Italy). A digital image of each embryo was taken, and the number of nuclei was counted.

#### Statistical analysis

All statistical analyses were performed using Stata/IC 11.2 (StataCorp LP, USA). In continuous data, normal distribution was checked by Shapiro-Wilk test. When the assumption of normality was met (follicles expressing > 3 γH2AX foci, ROS and GSH, blastocyst cell number) the data were analysed by one way ANOVA using Bonferroni as post-hoc test. When the data were not normally distributed (normal preantral follicles) a non-parametric Kruskal-Wallis test was used. Categorical data of meiotic progression, cleavage and development to blastocyst stage were analyzed by chi-square test. The level of statistical significance was set at *P* < 0.05.

## Results

### Experiment 1

#### Morphology of preantral follicles after ovary storage at 4°

A total of 998 preantral follicles were examined (mean ± SD for each group: 199.6 ± 24.3). The distribution of preantral follicles at each developmental stage was similar among storage groups. Most of the preantral follicles were at primordial stage (77.2 ± 1.2%), while primary (15.7 ± 1.1%) and secondary follicles (6.8 ± 1.2%) were represented in lower percentages.

**Fig. 1:**
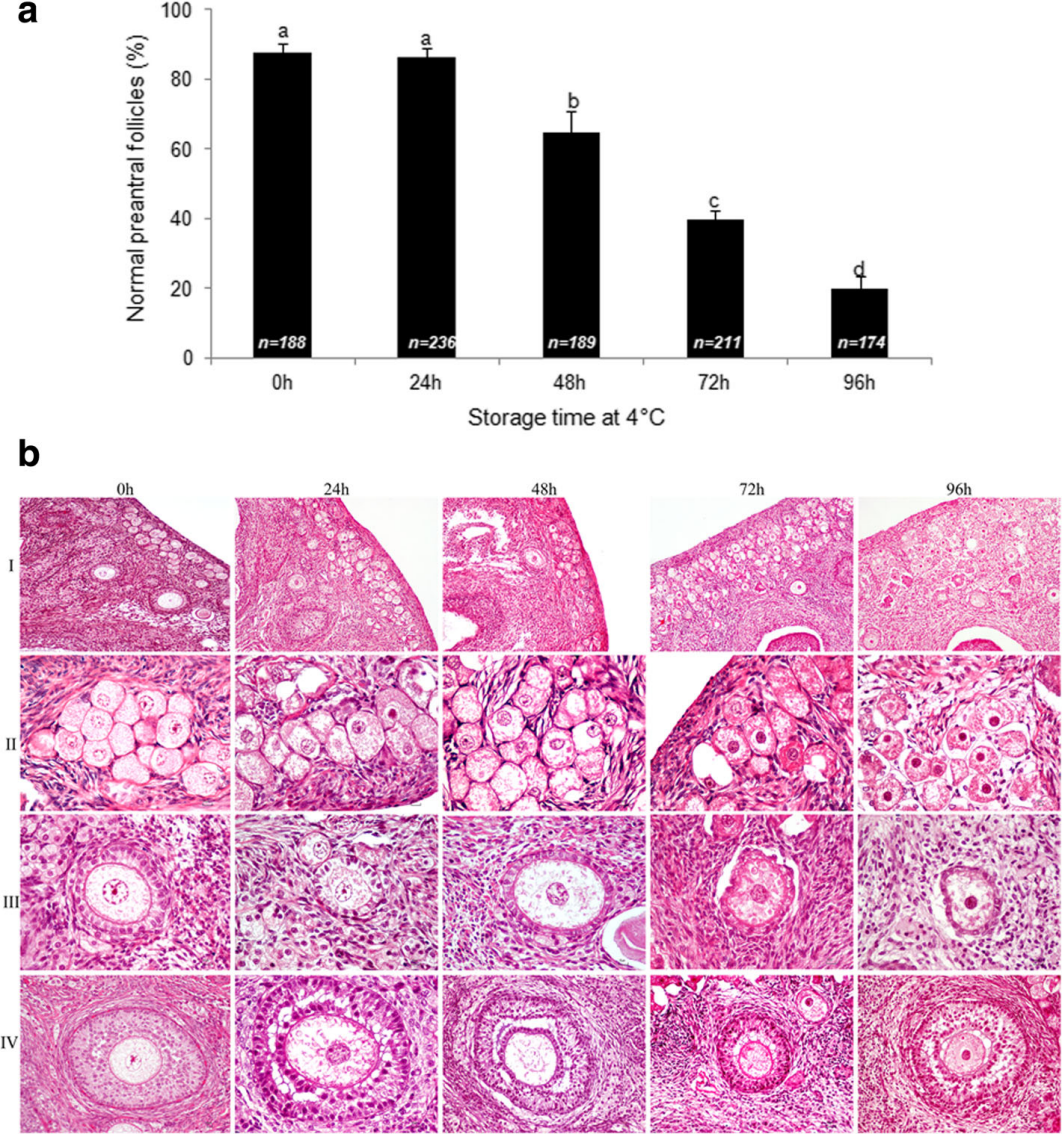
**a** Percentage (mean ± S.E.M) of morphologically normal preantral follicles after different time of ovary storage at 4 °C. **b** Representative images of sections of ovarian tissue (I) and different classes of preantral follicles (II-IV) stained by hematoxylin and eosin. Different superscripts (a, b, c, d) among storage times indicate *P* < 0.05. *n =* number of preantral follicles analyzed

The percentage of morphologically normal follicles at different storage times is shown in Fig. 1. Storage for 24 h maintained the proportion of follicles with normal morphology (86.3 ± 2.8%) similar to control values (87.7 ± 2.5%). A progressive decrease (*P* < 0.05) in the percentage of normal follicles was observed following 48 h (65.0 ± 6.1%), 72 h (39.7 ± 2.8%) and 96 h (20.0 ± 3.2%) of storage compared to 0 and 24 h groups.

#### Effect of ovary storage at 4 °C on the organization of the microtubular cytoskeleton

**Fig. 2:**
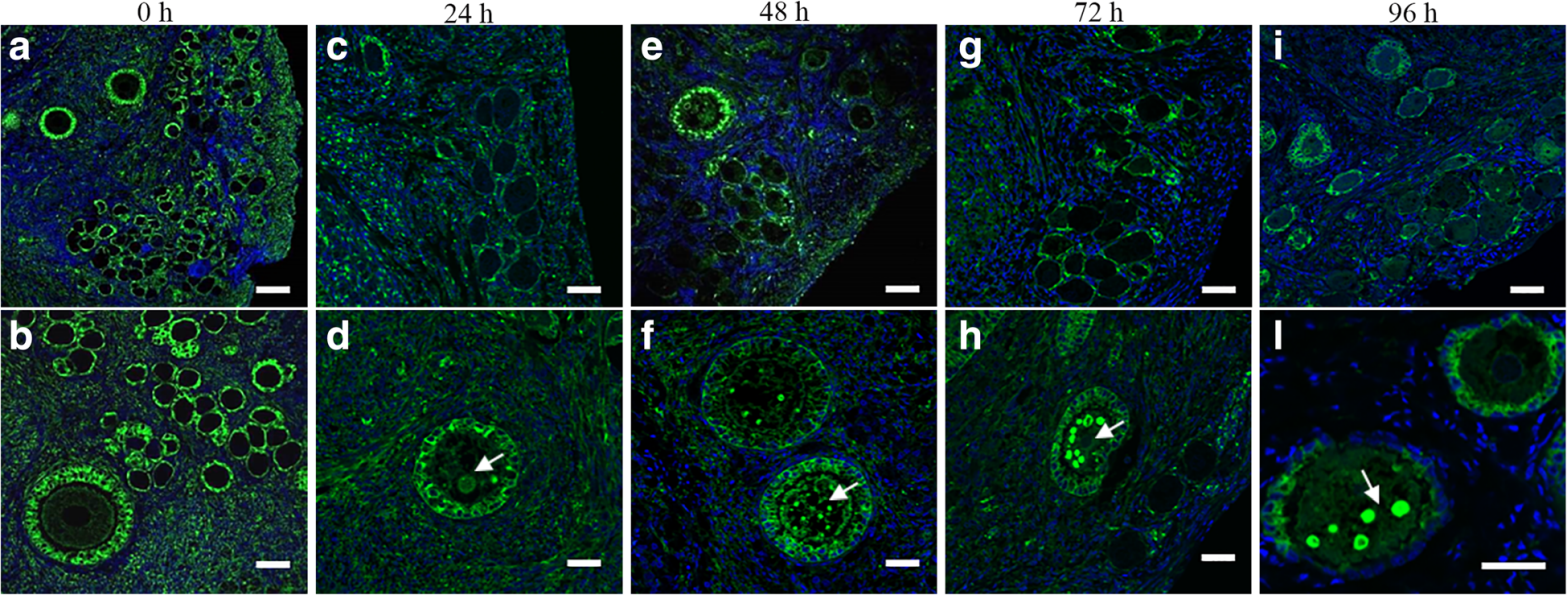
Representative images of tubulin organization of preantral follicles after different times of ovary storage at 4 °C. Tubulin staining of granulosa and stromal cells is progressively reduced with increasing storage time (**a**-**i**) and bright spots of tubulin appear in the cytoplasm of follicle enclosed oocyte (**d**-**l**, white arrow). Nuclei were stained with Hoechst 33,342 (blue). Scale bar = 20 μm (**a**, **c**, **e**, **g**, **i**)**;** 10 μm (**b**, **d**, **f**, **h**, **l**)

A storage time-dependent change of the tubulin organization was evident in cellular components of ovarian tissue (Fig. 2). In control sections, large amounts of tubulin were visible in the granulosa cell layers and a well detectable microtubular network was present throughout the cytoplasm of follicle-enclosed oocytes (Fig. 2 a, b). The tubulin distribution was progressively disrupted when increasing the storage time. Ovary storage at 4 °C for 24 h (Fig. 2 c,d) and 48 h (Fig. 2 e, f) caused a decrease in tubulin staining, which became irregularly distributed in granulosa and stromal cells. Storage for more extended periods (72 and 96 h) progressively induced tubulin alterations (Fig. 2 g,h,i, l). Similarly, the network of microtubules within the oocyte became irregular and disorganized following storage; spots of tubulin staining (see arrow in Fig. 2 f, h, and l**)** were observed inside the cytoplasm of the primary/secondary follicle-enclosed oocytes. The alterations in the tubulin network and the presence of tubulin spots in the oocyte cytoplasm were more evident when the storage time was prolonged.

#### Effect of ovary storage at 4 °C on the DNA integrity of preantral follicles

**Fig. 3:**
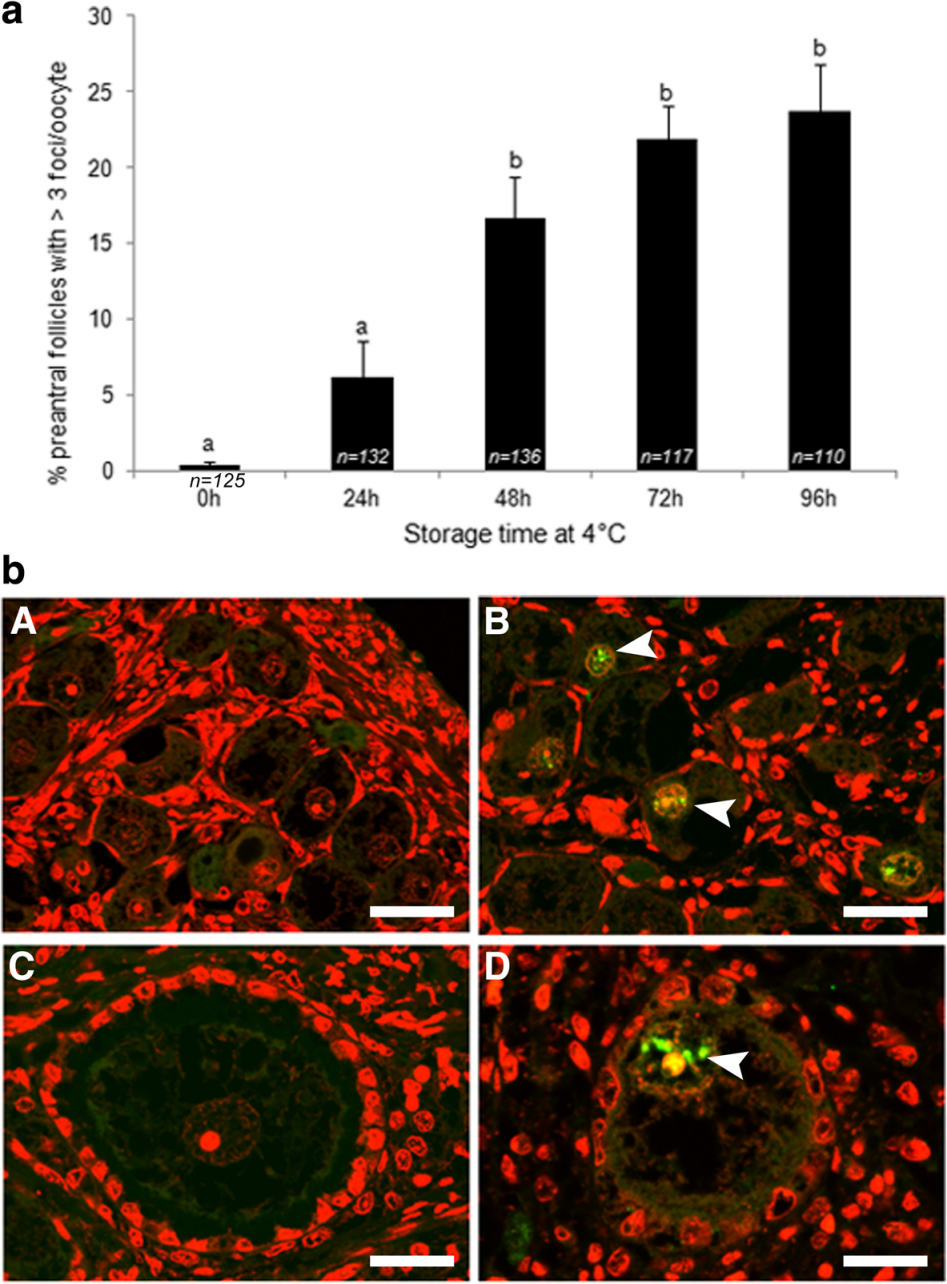
**a**. Representative images of fluorescent immunodetection of γH2AX phosphorylation: follicle-enclosed oocytes with nuclei without bright foci (A, C) or expressing green fluorescent foci (B, D, white arrows). **b** Percentage (mean ± S.E.M) of preantral follicles with 3 or more foci/oocyte after different time of ovary storage at 4 °C. Nuclei were stained with propidium iodide (red). Scale bar =10 μm. Different superscripts (a, b) among storage times indicate *P* < 0.05. *n* = number of preantral follicles analyzed per storage time

The presence of characteristic bright points (foci) within oocyte nuclei after immunostaining for γH2AX was considered to represent the induction of DNA DSBs. The percentage of follicles expressing distinct γH2AX foci and the number of foci they presented were assessed in preantral follicles after different times of ovary storage at 4 °C (Fig. 3). Since the control samples had very few oocytes with more than three foci (Fig. 3a, A, C), detection of three or more foci\nucleus was considered an indicator of DNA damage induced by ovary storage (Fig. 3a, B, D).

Low percentages of γH2AX-positive preantral follicles with > 3 foci/oocyte were observed in the 24 h stored group (6.2 ± 2.3) and did not differ from the control group (0.3 ± 0.3%). In contrast, γH2AX-positive foci were significantly (*P* < 0.05) more abundant in preantral follicles from ovaries stored for 48 h (16.6 ± 2.6%), 72 h (21.8 ± 2.1%) and 96 h (23.7 ± 3.1%) compared to control and 24 h stored groups (Fig. 3b).

No staining was observed in individual granulosa cells or in any other ovarian cell types.

### Experiment 2

#### Effect of ovary storage at 4 °C on the meiotic competence of oocytes

The in vitro meiotic progression of oocytes retrieved from antral follicles of ovaries stored at 4 °C for 0, 24, 48, 72 and 96 h is summarized in Table 1. A significantly higher (*P* < 0.05) number of oocytes in the 96 h group stayed at the GV stage compared to the other groups. The percentage of oocytes that resumed meiosis (GVBD/ MI stage) did not differ among the five groups. The percentages of oocytes reaching MII stage were similar among 0, 24 and 48 h stored groups. A significantly lower (*P* < 0.05) MII rate was detected following storage for 72 and 96 h. The degeneration rate significantly increased with storage time (*P* < 0.05).

**Table 1:** In vitro maturation of cat oocytes retrieved from ovaries stored for different times at 4 °C

Nuclear configuration after IVM					
Storage time (h)	n° oocytes	GV (%)	GVBD/MI (%)	MII (%)	Deg (%)
0	109	25 (22.9%)^a^	14 (12.8%)	67 (61.5%)^a^	3 (2.8%)^a^
24	114	24 (21.1%)^a^	8 (7.0%)	70 (61.4%)^a^	12 (10.5%)^b^
48	83	11 (13.2%)^a^	4 (4.8%)	58 (69.9%)^a^	10 (12.1%)^b^
72	58	16 (27.6%)^a^	2 (3.5%)	19 (32.7%)^b^	21 (36.2%)^c^
96	62	28 (45.2%)^b^	4 (6.5%)	11 (17.7%)^b^	19 (30.6%)^c^

*GV* germinal vesicle, *GVBD* germinal vesicle breakdown, *MI* metaphase I, *MII* metaphase II, *Deg* degenerated

Different superscripts (a, b, c) *P* < 0.05

#### Effect of ovary storage at 4 °C on intracellular levels of GSH, ROS, and lipid peroxidation of in vitro matured oocytes

**Fig. 4:**
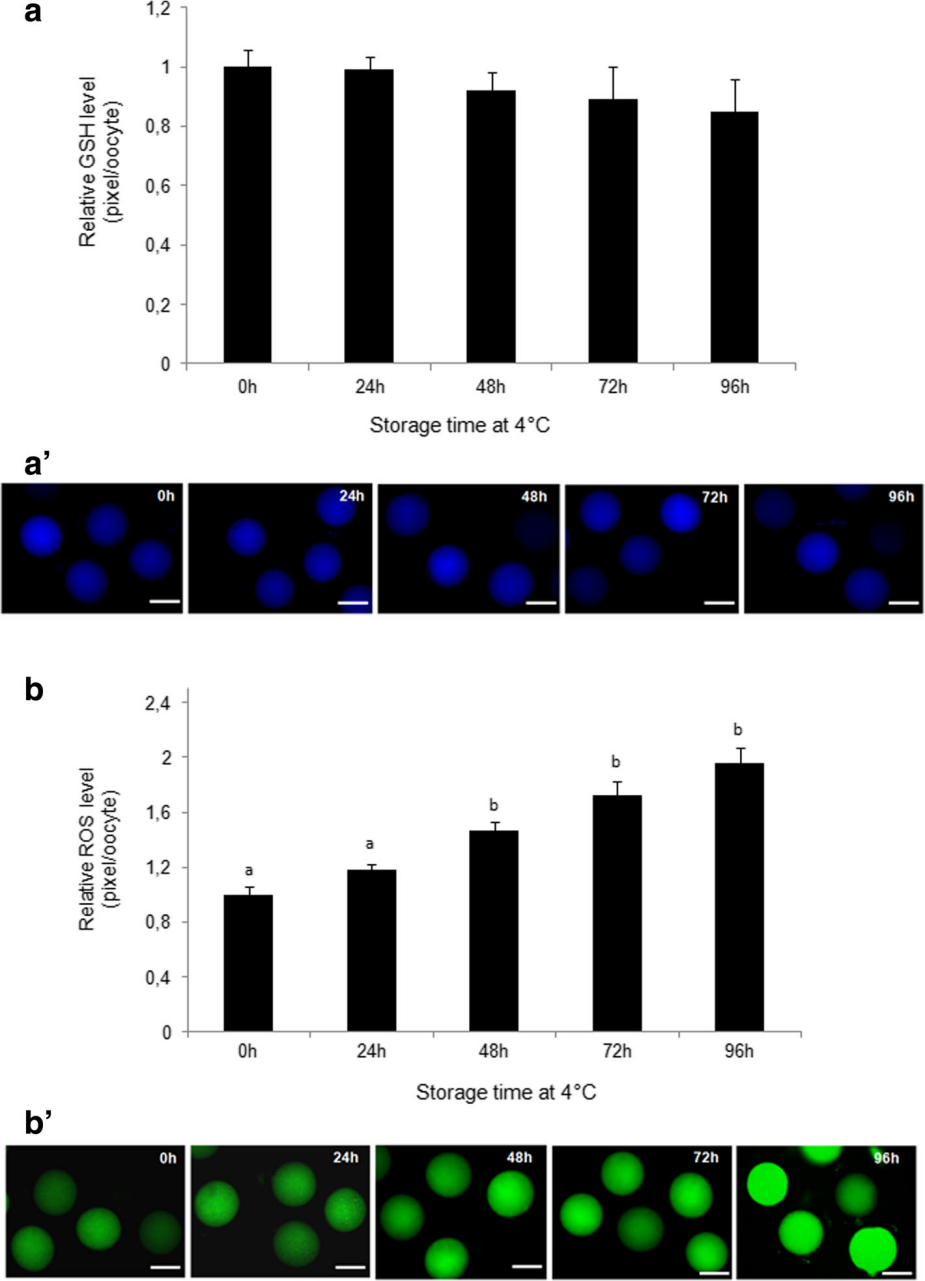
Effect of ovary storage time at 4 °C on intracellular GSH (**a**) and ROS levels (**b**) of in vitro matured oocytes. Epifluorescence photomicrographs of MII oocytes that were stained with CellTracker Blue to determine the level of GSH (**a’**) and with 2′7′-dichlorodihydrofluorescein diacetate (H2DCFDA) to detect ROS (**b’**). Different superscripts (a, b, c, d) among storage times indicate P < 0.05. Scale bar =50 μm

The intracellular level of GSH was not statistically different among groups (Fig. 4a, a’).

A significantly higher level (*P* < 0.05) of ROS was recorded in in vitro matured oocytes recovered from ovaries stored for 48, 72 and 96 h compared to oocytes obtained from ovaries stored for 0 and 24 h (Fig. 4b, b’).

**Fig. 5:**
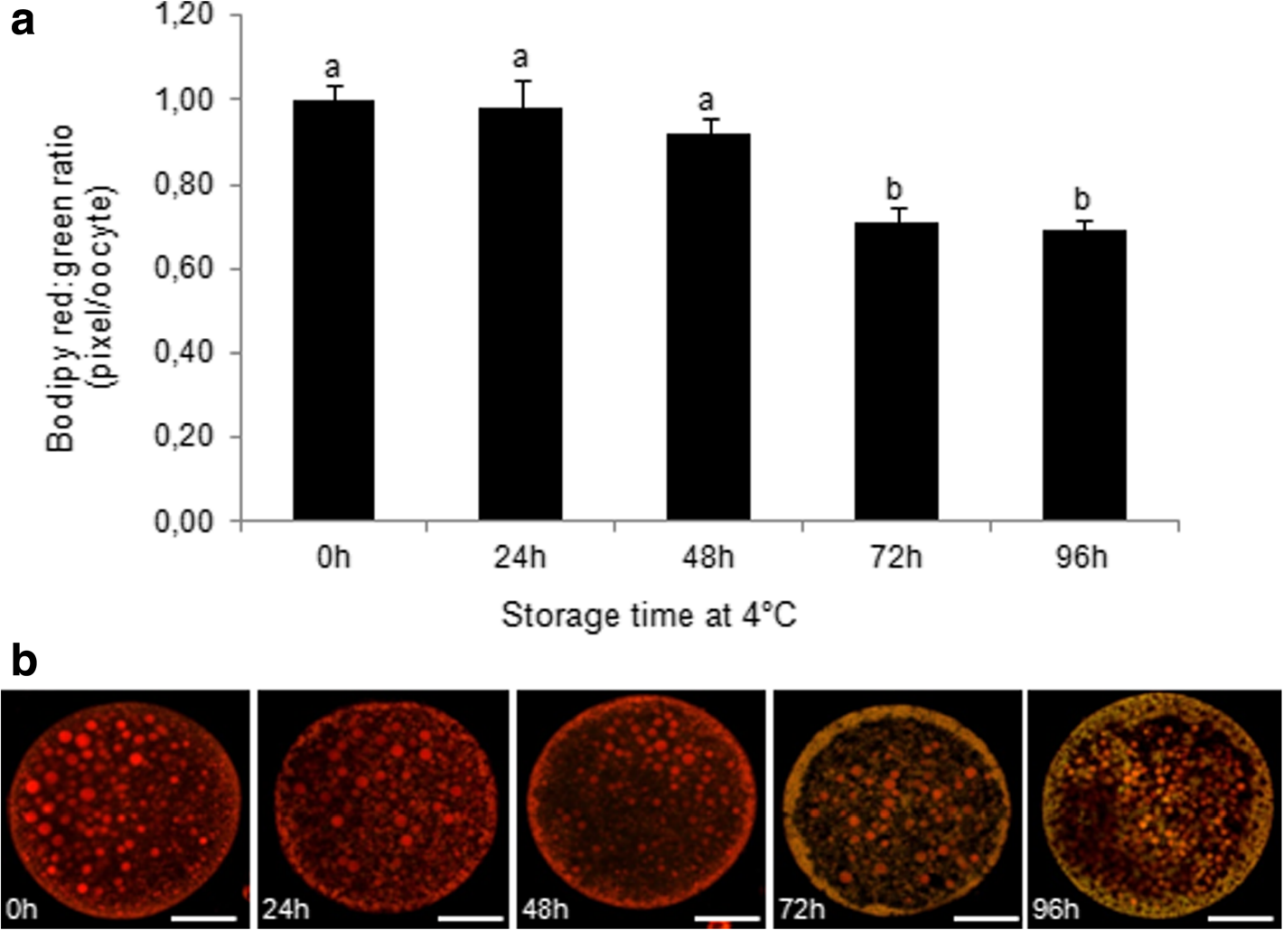
**a** Levels of lipid peroxidation as measured by the ratio of red: green fluorescence of in vitro matured oocytes recovered from ovaries stored at 4 °C for different times. **b** Epifluorescence photomicrographs of matured oocytes that were stained with BODIPY. Scale bar =100 μm. Different superscripts (a, b) among storage times indicate *P* < 0.05.

The levels of lipid peroxidation of in in vitro matured oocytes retrieved from ovaries stored for 72 and 96 h were higher than those in oocytes from 0, 24 and 48 h groups (Fig. 5a, b).

#### Effect of ovary storage at 4 °C on oocyte developmental competence

**Fig. 6:**
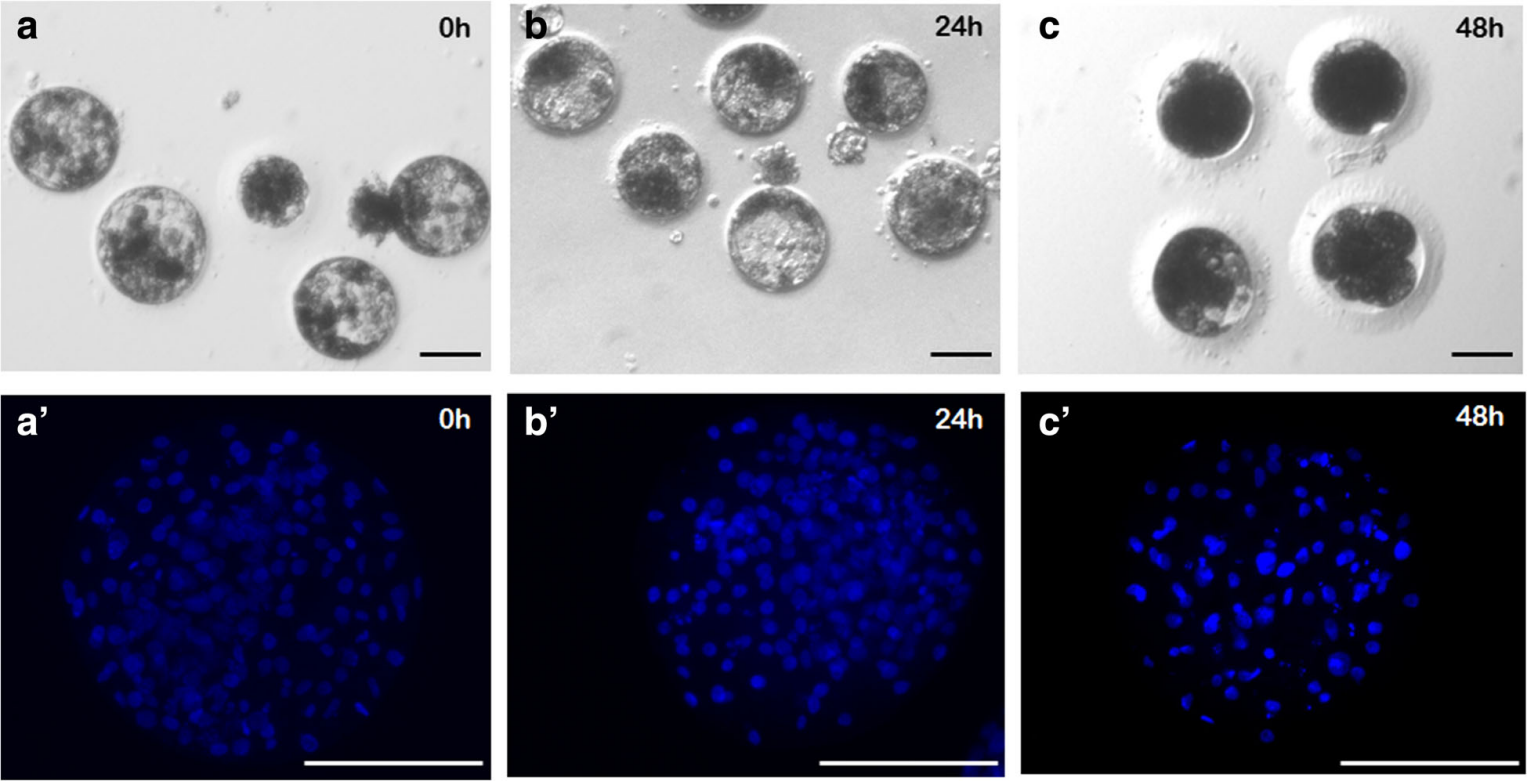
In vitro produced blastocysts obtained after in vitro maturation and fertilization of oocytes recovered from ovaries stored for 0, 24 and 48 h at 4 °C under phase contrast microscopy (**a**, **b**, **c**) and under epifluorescent microscopy after staining with Hoechst 33,342 (**a’**, **b′**,**c′**). Scale bar =100 μm

The proportion of oocytes that progressed to the first cleavage stage after in vitro fertilization was similar among 0, 24 and 48 h stored groups. A significantly (*P* < 0.05) decrease of cleavage rate was observed following 72 and 96 h of ovary storage (Table 2). The percentages of blastocysts relative to the number of cleaved embryos (developmental competence) and to the total number of oocytes (blastocyst yield) were lower in 48, 72 and 96 h stored groups compared to those in 0 and 24 h groups (Table 2, Fig. 6). Cell numbers in blastocysts from 0 and 24 h groups were similar. Only one blastocyst with 98 cells developed after 48 h of ovary storage.

**Table 2:** In vitro embryo development after in vitro fertilization of cat oocytes retrieved from ovaries stored for different times at 4 °C

Storage time (h)	n°oocytes fertilized	n°cleaved embryos	n°blastocysts/ cleaved/embryos	n°blastocysts/ total oocytes	Blastocyst cell number (mean ± SD)
0	56	25 (44.6%)^a^	15 (60%) ^a^	15 (26.8%) ^a^	178.5 ± 40.5 ^a^
24	48	19 (39.6%) ^a^	11 (57.9%) ^a^	11 (22.9%) ^a^	171.9 ± 44.6 ^a^
48	43	16 (37.2%) ^a^	1 (6.2%) ^b^	1 (2.3%) ^b^	98 ^b^
72	41	2 (4.9%)^b^	0 (0%) ^b^	0 (0%) ^b^	–
96	39	0 (0%) ^b^	0 (0%) ^b^	0 (0%) ^b^	–

Different superscripts (a, b) *P* < 0.05

## Discussion

This study is the first to examine the effect of cat ovary storage at 4 °C for an extended period of time (up to 96 h) on the morphology, microtubular cytoskeleton and DNA damage in preantral follicles. We also investigated the in vitro meiotic and developmental competence of oocytes recovered from antral follicles and parameters of oxidative stress as indicators of the quality of matured oocytes. The main findings of the research were: a) storage up to 24 h did not affect the morphology and DNA integrity of preantral follicles, whereas the organization of the microtubular cytoskeleton was moderately altered; b) extended storage times (48, 72, 96 h) caused progressive structural changes of preantral follicles as showed by morphological abnormality, disassembling of microtubules and DNA damage; c) ovary storage up to 48 h did not influence the in vitro meiotic maturation ability of the oocytes retrieved from antral follicles but only the oocytes stored within the ovary for 24 h developed to blastocyst stage in percentage similar to control oocytes; d) the duration of storage did not affect the GSH levels, whereas increased levels of ROS were detected starting from 48 h and lipid peroxidation at 72 and 96 h of storage.

Hypothermic storage (0–7 °C) of organs is normally used to minimize tissue metabolism and prevent ischemic injury, and the maximum storage time depends on the specific organs. Several studies described transportation at cold temperature of mammalian, including human, ovarian tissue for fertility preservation. Indeed, the transportation of human ovarian tissue at low temperature and within a period of 4 to 26 h didn’t affect human ovarian tissue morphology, viability, and follicle development in vitro and in vivo [53–57]. On the other hand, a study by Klocke et al. [58] investigating the effects that storage at 4 °C for 24 or 48 h before vitrification has on the human ovarian cortex highlighted significant negative consequences within 24 h.

Some investigators [55, 56, 59] recommended cooling human ovarian tissue to low positive temperatures before cryopreservation independently of transportation purposes. The Storage of human ovarian cortex for 24 h at 5 °C before cryopreservation increased the viability of the follicles after thawing and in vitro culture in the chorioallantoic membrane system [56]. Moreover, the same group also showed, after in vitro culture, a significant increase of neo-vascularisation in human ovarian tissue pre-cooled at 5 °C for 24 h compared to non-cooled control tissue [55] and a reduced translocation of phosphatidylserine, a membrane component with a key role in the mechanisms of necrosis and apoptosis [59].

Certainly the proof of the efficiency of ovarian tissue storage at cold temperatures during transportation are the successful births reported in European countries where ovarian transportation for long distance is conducted [29, 60–63].

Evidence from animal studies has shown that chilling ovarian tissue at 4 °C during transportation provides optimal conditions to preserve follicular morphology and viability for longer times than at higher temperatures. In the mouse, storage at 4 °C for up to 24 h has no deleterious effect on histological morphology of ovarian tissue or on mature gametes [27]. Similarly, ovarian follicles from sheep and goat [23, 64] were successfully preserved for up to 24 h and ovarian follicles from zebu cows for up to 18 h [65]. In the canine [24] and equine models [25], the morphology of preantral follicles was best maintained when ovaries were stored at 4 °C for shorter periods (12 and 4 h respectively).

Our study clearly demonstrated that morphological changes in the preantral follicle population of cat ovaries stored at 4 °C was modest for at least 24 h, maintaining about 86.3% of morphologically normal follicles. Longer storage times caused a progressive damage to preantral follicles as shown by the percentages of normal follicles following 48 h (65%), 72 h (39.7%) and 96 h (20%) of storage.

Vitrification is an ideal option for preserving ovarian tissue especially for wildlife species because it doesn’t require specialized equipment and can be performed in the field. To date, there are only few studies on vitrification of feline ovarian tissue [66–71] and, despite recent progress, this procedure still requires modification and optimization. The percentage of vitrified-warmed morphologically normal follicles, immediately after warming, ranged from 5.9% [66] to a maximum 58.8% [69] under the optimal conditions. The storage at 4 °C can be a useful method for short-term preservation of ovarian tissue because the normal morphology of preantral follicles remains high even after 48 h storage, allowing xenotrasplantation into recipient animals or in vitro culture of follicles.

Information regarding the influence of cold storage on preantral follicles of cat ovaries is scarce. A study by Cocchia et al. [12] explored cell viability in cat ovaries stored at 4 °C up to 72 h and reported a storage time-dependent increase in the percentage of apoptotic areas in the ovarian tissue. Recently, the impact of holding of cat ovaries at 4 °C for 24 and 72 h followed by vitrification has been investigated by monitoring the survival and morphology of preantral follicles with neutral red staining, histology and ultrastructural analysis by transmission electron microscopy [22]. Results indicated a reduction in the number of intact and viable follicles after 24 and 72 h storage without significant differences between groups and time points. As reported by the Authors, the large variation in the number of follicles evaluated and the high standard deviations probably masked differences between groups and time points. The variability of sample numbers as well as differences in experimental design and methodology make it difficult to compare these data with our results. Indeed, cortical slices of ovaries stored for 24 and 72 h were incubated for 16 h with neutral red before viability and morphology assessment. The small number of follicles utilized for transmission electron microscopy affected the accuracy of the ultrastructural evaluation, although it revealed the preservation of subcellular structures after cooling for 24 h. Authors finally concluded that the ovarian tissue can be maintained for a maximum of 24 h at 4 °C before cryopreservation.

A further goal of our study was to investigate the response of the microtubular cytoskeleton of preantral follicles to chilling at 4 °C. The presence of an intact cytoskeleton and its dynamic organization are crucial during folliculogenesis and oogenesis [72]. The effect of cold temperature is known to induce depolimerization of microtubules [73] in mammalian cells. Chilling injuries on meiotic spindles of fully grown oocytes have been extensively reported in several species [74–76]. A previous study on mice [42] investigated the effect of chilling at 0 °C on the cytoskeleton organization of isolated early preantral follicles. The results showed that chilling for only 1 min was sufficient to cause depolymerization of microtubules in the oocytes and the surrounding granulosa cell layer. In the present study, an alteration of microtubular network in preantral follicles of ovaries stored at 4 °C for different times has been detected. Observed changes included tubulin depolimerization and disassembling in the granulosa cell layer and the appearance of tubulin spots in the oocytes of preantral follicles. The extent of microtubule modifications depended on the time of ovary storage: these started from 24 h of storage and became more evident following 48, 72 and 96 h. Further studies are needed to understand the reversibility of the cooling effect on the microtubular cytoskeleton. Preliminary evaluations (data not shown) indicated that the microtubule modification was fully reversible when ovaries stored at 4 °C for 24 and 48 h were re-warmed at 38.5 °C for 2 h.

Beside the morphological and structural analysis of ovarian tissue, the preservation of DNA integrity is fundamental in order to understand the effect of low temperature on preantral follicles. DNA double-strand breaks in oocytes enclosed in preantral follicles have never been investigated after storing of cat ovaries at 4 °C. Cocchia et al. [12] previously evaluated apoptosis by TUNEL assay in the cat ovarian tissue stored at 4 °C up to 72 h and indicated that the addition of the antioxidant enzyme superoxide dismutase (SOD) to transportation medium reduced cellular apoptosis, albeit these differences were not statistically relevant. Double-strand breaks of DNA (DSBs) are a marker of severe DNA damage, which can activate apoptosis [45] if not repaired. We have demonstrated that ovary storage at 4 °C for over 24 h induces DSBs in oocytes enclosed in preantral follicles and that DNA damage increases as storage is prolonged. Additional analysis is needed to assess the DNA repair capability of oocytes to ultimately confirm the exact extent of the DNA damage.

Our explorative study focuses mainly on the evaluation of morphological and structural modifications in preantral follicles in feline ovarian tissue after storage at 4 °C for a prolonged period. The next step should be to validate the quality and survival of stored ovarian tissue by assessing the developmental competence of the preantral follicles following xenotrasplantation of ovarian cortex into immunodeficient recipients or after in vitro culture.

The second part of our study was aimed to determine the adverse effects of cold storage on the oocytes harvested from antral follicles by analyzing their meiotic and developmental competence after in vitro fertilization.

Our findings indicated that when ovaries were stored at 4 °C for 24 and 48 h, oocytes retained the ability to progress to metaphase II at a rate similar to control oocytes, while a significant reduction of nuclear maturation was registered after 72 and 96 h of storage. The storage of ovarian tissue at 4 °C (at least for 48 h) may offer the possibility to preserve viability and meiotic competence of oocytes retrieved from antral follicles otherwise compromised by vitrification of ovarian tissue. Indeeed, Luvoni et al.*..* [66] reported that oocytes recovered from vitrified ovarian tissue showed higher degeneration rate and lower rate of meiosis resumption compared to those collected from fresh tissue.

Our data corroborate the results previously reported by other Authors [4, 6, 9], showing that ovary storage at 4 °C for 24 and 48 h has no effect on the in vitro meiotic competence of cat oocytes. Conversely, Evecen et al. [77] documented that, while storing ovaries for 24 h at 4 °C did not affect the meiotic competence of oocytes in vitro, 48 h of storage decreased it dramatically. Unaffected IVM rates were reported when cat ovaries were stored at 4 °C up to 72 h; however, only oocytes stored within the ovary for 24 h could produce blastocysts after in vitro fertilization [9, 10]. These developed into live kittens after transfer to recipients [10]. Similarly, our results showed that cleavage frequency, in vitro development to blastocyst stage and blastocyst cell number did not were not different after recovery of oocytes from fresh and 24 h stored ovaries. Moreover, we observed that the developmental competence of oocytes declined markedly after 24 h of storage. Recently, Cocchia et al [12] showed that SOD supplementation in the transportation medium of domestic cat ovaries enhances embryo production after 48 and 72 h of storage.

Our rates of blastocyst formation in the 24 h stored group (22.9%) are higher than those obtained after in vitro maturation of cat oocytes in presence of relaxin [11] or with the addition of SOD during ovary storage [12].

One of the main factors contributing to the poor quality of in vitro matured oocytes may be oxidative stress (OS). Under physiological conditions the antioxidant defense mechanism of oocytes and ovaries, consisting of enzymatic antioxidants such as superoxide dismutase (SOD), catalase (CAT) and glutathione peroxidase (GPx) and non-enzymatic antioxidants (GSH), detoxifies excess ROS maintaining the oxidant/antioxidant balance. However, increased levels of ROS beyond the physiological range may lead to OS and cause a wide range of molecular damages, including lipid peroxidation and protein and DNA damage resulting in deterioration of oocyte quality [78, 79]. The ROS scavenging ability of oocytes decreases under pathological conditions, different types environmental stress, and aging or elevated oxygen tension during in vitro manipulation [80, 81]. Glutathione is the major non-protein sulphydryl compound in mammalian cells and protects them from oxidative damage [82]. The level of GSH in oocytes increases as the oocyte resumes meiosis, and higher concentrations are found in mature oocytes than in immature ones [83–85].

Several studies underlined that the GSH content in oocytes at the end of IVM appears to reflect the degree of cytoplasmic maturation and quality of oocytes [86–89].

Our results showed that when ovaries were stored for different times at 4 °C the content of intracellular GSH of in vitro matured oocytes was slightly lower than that in control oocytes, but without statistical difference among groups. We speculate that, given the availability of cysteine in the maturation medium, GSH may be properly synthesized as long as the oocytes retain the ability to complete maturation. A previous study provided evidence that the availability of constitutive amino acids, in particular cysteine, is the limiting factor for GSH synthesis in mammalian oocytes [87].

Beside constant levels of GSH, the analysis of the selected oxidative stress markers in matured oocytes showed a progressive increase in ROS levels from 48 h of ovary storage associated with elevated lipid peroxidation at 72 and 96 h of storage. Lipids are highly susceptible to the attack of ROS [90] and their peroxidation may damage the oocyte membrane, modifying its structure and function.

Long-term storage of ovaries at 4 °C (72 and 96 h) may have altered the activity of other enzymes which are involved in modulating the antioxidant defense system in oocytes during in vitro maturation. Further evaluation is necessary to improve knowledge on the redox state of oocytes recovered from cold stored ovaries.

In light of these results, we suggest that oxidative stress is one of the factors hindering the developmental capacity of in vitro matured oocytes following ovary storage for more than 24 h. Strategies aimed at reducing oxidative stress, including the addition of antioxidants in the oocyte maturation medium or in the transportation medium of the ovaries, may improve oocyte quality.

Collectively, the results provide additional information on the effect of cold storage on preantral follicles and on oocytes from antral follicles and may help in setting up programs for fertility conservation, especially for wild feline species which die in geographic areas located far from ARTs centers.

## Conclusions

In summary, the results of our study revealed that morphological and structural changes of preantral follicles are minimal after storage for up to 24 h. These alterations progressively increase from 48 h onwards and become more severe at 72 and 96 h of storage. The extension of the storage period beyond 24 h negatively influences the oxidative status of oocytes after in vitro maturation and their developmental competence.
